# Fetal dose assessment in a pregnant patient with brain tumor: A comparative study of proton PBS and 3DCRT/VMAT radiation therapy techniques

**DOI:** 10.1002/acm2.14394

**Published:** 2024-06-17

**Authors:** Robabeh Rahimi, Michael Taylor, Xing Li, Kuan Ling Chen, Grayden MacLennan, Erin Murdoch, Lienard Chang, Ashkan Parniani, Peng Wang, Ashish Chawla, Jiajin Fan, Daniel Kim

**Affiliations:** ^1^ Radiation Oncology Department Inova Health System Fairfax Virginia USA

**Keywords:** 3D conformal radiation therapy, brain tumor, fetal dose, neutron dose, pencil beam scanning, proton therapy, re‐irradiation, volumetric‐modulated arc therapy

## Abstract

**Purpose:**

The treatment of brain tumors in pregnant patients poses challenges, as the out‐of‐field dose exposure to the fetus can potentially be harmful. A pregnant patient with prior radiation treatment was presented with a brain tumor at our clinic. This work reports on our pre‐treatment study that compared fetal dose exposure between intensity‐modulated proton therapy (IMPT) using pencil beam scanning (PBS) and conventional photon 3D conformal radiation therapy (3DCRT) and volumetric‐modulated arc therapy (VMAT), and the subsequent pregnant patient's radiation treatment.

**Materials and methods:**

Pre‐treatment measurements of clinical plans, 3DCRT, VMAT, and IMPT, were conducted on a phantom. Measurements were performed using a device capable of neutron detections, closely following AAPM guidelines, TG158. For photon measurements, fetus shielding was utilized. On patient treatment days, which was determined to be proton treatment, shielding was used only during daily imaging for patient setup. Additionally, an in vivo measurement was conducted on the patient.

**Results:**

Measurements showed that IMPT delivered the lowest fetal dose, considering both photon and neutron out‐of‐field doses to the fetus, even when shielding was implemented for photon measurements. Additionally, the proton plans demonstrated superior treatment for the mother, a reirradiation case.

**Conclusion:**

The patient was treated with proton therapy, and the baby was subsequently delivered at full term with no complications. This case study supports previous clinical findings and advocates for the expanded use of proton therapy in this patient population.

## 1 INTRODUCTION

Radiation therapy for managing cancer during pregnancy carries the potential risk of fetal radiation exposure. Radiation dose exposure to the fetus can result in miscarriage, fetal growth restriction, mental retardation, functional impairments, a reduction in IQ, and an increased risk of future cancers.^1^, ^2^, ^3^, ^4^ Comprehensive risk‐benefit assessments should guide treatment decisions, involving the patient and the care team.

The American Association of Physicists in Medicine (AAPM) has issued guidelines concerning fetal dose from radiation therapy and its impact on the patient's fetus. The impacting factors include gestational stage and the radiation dose received by the fetus. For example, a radiation dose exceeding 0.5 Gy is associated with high risks of damage during all trimesters, while a radiation dose ranging from 0.1  to 0.5 Gy is considered harmful during the first trimester.^5^, ^6^, ^7^, ^8^

Minimizing fetal exposure to radiation is essential while ensuring effective cancer treatment for the mother. The radiation dose to the fetus depends on variables such as the distance of the fetus from the field edge, planning techniques, delivery methods, technology utilization (e.g., shielding), and the choice between photon and proton radiation. Out‐of‐field radiation in photons primarily consists of patient scatter, collimator scatter, and gantry head leakage. When using a low‐energy photon beam, typically below 10 MV, the out‐of‐field radiation contains negligible neutron components. In proton treatment, conversely, the primary source of out‐of‐field radiation originates from neutrons generated along the beam path and within gantry accessories, as well as inside the patient's body. In this context, Pencil Beam Scanning (PBS) proton delivery systems are superior to scattering systems in terms of minimizing the out‐of‐field neutrons originating from sources outside the patient's body, although in‐patient‐generated neutrons remain a concern.^9^

Photon‐based radiation therapy techniques like 3D conformal radiation therapy (3DCRT) and volumetric‐modulated arc therapy (VMAT) have been used for the treatment of pregnant patients with brain tumors.^8^, ^9^, ^10^, ^11^ Proton therapy is noteworthy for its ability to minimize radiation exposure to healthy tissues, making it a preferred option for patients requiring reirradiation.^12^ Whether using photon or proton treatment, it is important to note that currently existing commercial Treatment Planning Systems (TPS) lack the capability to accurately model out‐of‐field dose, including photon scatter and neutron contributions, accurately. Consequently, measurement becomes the ultimate solution for estimating out‐of‐field doses.^13^

Measuring the photon components of the out‐of‐field dose can be accomplished accurately using a variety of small, sensitive detectors that have been proven practical and effective for this specific purpose. However, measurement of neutron out‐of‐field dose presents a more complex challenge for several reasons.^8^

Neutrons generated during proton therapy encompass a wide energy range, spanning from thermal energies, that is, below eV, to the highest energy of the protons, potentially exceeding 200 MeV. This diverse energy spectrum necessitates the consideration of different Relative Biological Effectiveness (RBE) factors for estimating the final dose. Furthermore, neutrons can travel long distances through the medium due to their neutral charge, and owing to their broad energy spectrum, the out‐of‐field dose is being spread over a wide spatial range. Given that the neutron out‐of‐field dose is typically at a low‐level magnitude, it is imperative to utilize highly sensitive detectors capable of effectively capturing a broad spectrum of neutron energies, and with well‐defined RBE factors. While conventional bulky neutron detectors can be employed for detecting this broad energy range, they should be used cautiously as their large size can potentially influence the existing neutron field.^14^, ^15^, ^16^, ^17^, ^18^, ^19^

Measurements represent the definitive method for estimating out‐of‐field neutron dose; however, practical implementation of out‐of‐field dose measurement can be resource‐intensive and equipment demanding. This poses challenges, particularly for the small proton therapy centers that are increasingly emerging. Hence, it is crucial to underscore the importance of collaborative case studies and the collective compilation of a comprehensive database, which ultimately benefits all patients.^20^, ^21^, ^22^, ^23^, ^24^, ^25^

This case study reports the results of the conducted pre‐treatment study that involved a comparative analysis of PBS IMPT, photon 3DCRT, and VMAT for a pregnant patient with prior radiation who received proton treatment in our center.

## 2 MATERIALS AND METHODS

Radiation therapy was prescribed for a 33‐year‐old pregnant patient diagnosed with astrocytoma, who had prior radiation to the same site. Simulations were conducted using a GE Revolution CT scanner and a 3T (Tesla) GE Architect MRI simulator. The use of an adjunct MR simulation scan, subsequently fused to the CT image to assist in delineating the target volumes, obviated the need for a thicker slice thickness on the CT simulation, thereby further reducing the radiation exposure to the fetus during the CT imaging process. Additionally, the CT scan region was deliberately confined to the target area, and lead aprons were positioned around the patient's abdomen and pelvis, all attempting to minimize the dose from imaging to the fetus.

Multiple treatment plans were developed, incorporating both photon and proton therapy modalities. Photon plans used 6MV beam energy. 3DCRT plans with various combinations of gantry and couch angles were generated. VMAT plans were devised, considering options such as uniform beams and flattened filter‐free (FFF) beams, as well as partial gantry rotations and controlled modulation. The proton plans explored various combinations of gantry and couch angles, with and without the use of a range shifter, and control on the highest energy layer in the plan.

In the case of photon pre‐treatment measurements, the shielding was carefully designed and employed to mitigate both gantry head leakage dose and the collimator scatter dose to the fetus.^7^, ^15^, ^26^ However, for the proton pre‐treatment study, no shielding was utilized because the fetal dose primarily resulted from neutrons generated within the patient's body during treatment.

All pre‐treatment measurements were conducted using a combination of an anthropomorphic phantom, featuring removable layers spaced at 3.8 cm (1.5‐inch) intervals, and solid water. This setup was chosen to closely replicate the size and characteristics of the pregnant patient's body, facilitating accurate measurements. Several measurements were taken at various points corresponding to distances resembling the positions of the fetus relative to the field edge.

Optically stimulated luminescent dosimeters (OSLDs), calibrated within our department for photon beam applications, were employed to assess the out‐of‐field dose to the fetus during pre‐treatment measurements with photon plans.^27^ For proton plans, the assessment of the out‐of‐field dose to the fetus was conducted using a calibrated Wide‐Energy Neutron Detection Instrument (WENDI‐II) manufactured by Thermo Fisher Scientific (Waltham, Massachusetts, USA),^28^, ^29^, ^30^ and small‐sized Landauer Neutrak neutron detectors.^6^ We utilized Luxel+ badge detectors from Landauer, ensuring they were not among the recalled detectors (Nanodots and Microstarii Reader).

The WENDI‐II detector is suitable for the wide range of energy detection of neutrons with high sensitivity. However, it is a large‐size detector, and its placement inside the phantom can potentially alter the phantom shape, deviating the measurement setup from a clinical case. Neutrak detectors were preferred over WENDI‐II due to their smaller size and the possibility of their use at multiple locations on and inside the phantom. Proton plans were anticipated to predominantly generate fast neutrons, to which Neutrak detectors exhibited limited sensitivity. AAPM TG158 Figure 2, ^8^ shows that, uniquely to proton, the neutron spectra have a second peak that starts at around 20 MeV and extends up to the maximum proton beam energy (172 MeV in Figure 2 AAPM TG158), meaning that in our case with the maximum energy in the proton plan of 120 MeV, and considering the Neutrak sensitivity that is up to 40 MeV, a correction factor should be applied to the Neutrak readings, and we chose a 10‐fold correction factor which was a conservative estimation, preferred for the safety of both the patient and the fetus.

### 2.1 Treatment planning

The treatment plans were created using the patient's CT scan data. VMAT and IMPT plans were developed using RayStation version 11A (RaySearch Laboratories, Stockholm, Sweden), with VMAT utilizing Collapsed Cone and IMPT employing Monte Carlo algorithms. The 3DCRT plan was created using Varian Eclipse 16.1 employing the analytical anisotropic algorithm (AAA). All treatment plans were designed to deliver a total dose of 4000 cGy in 15 fractions, with the prescription dose targeted to cover 95% of the target volume. Various planning techniques were explored to optimize the patient's treatment while simultaneously minimizing the out‐of‐field dose to the fetus.

In the case of photon plans, the beam isocenter was located superiorly and outside the patient's body to ensure the maximum achievable distance of the gantry head from the fetus. This arrangement also ensured sufficient clearance to prevent potential collisions between the gantry and the shielding setup when lateral beams were used. In the VMAT plan, an effort was made to maintain a low monitor unit (MU) to minimize the gantry head leakage dose.

A flattening filter‐free (FFF) beam, unlike a uniform beam, generally reduces the out‐of‐field dose due to its lower target current and the absence of a high‐scatter medium in the beam path. However, it is important to note that an FFF beam may also increase the out‐of‐field dose due to its softer nature, leading to additional dose contributions from low‐energy scatter photons. In the context of this specific case, it was determined that using a FFF beam would be advantageous since, based on the fetus's proximity to the field edge, it was highly unlikely for low‐energy scatter photons to reach the fetus, thus mitigating the potential dose increase associated with an FFF beam.

In the case of proton plans, various combinations of gantry and couch angles were explored. The use of a range shifter was considered. Additionally, different plan configurations, such as varying the number of beams and adjusting plan parameters like the highest energy in the plan, were examined.

**FIGURE 1:**
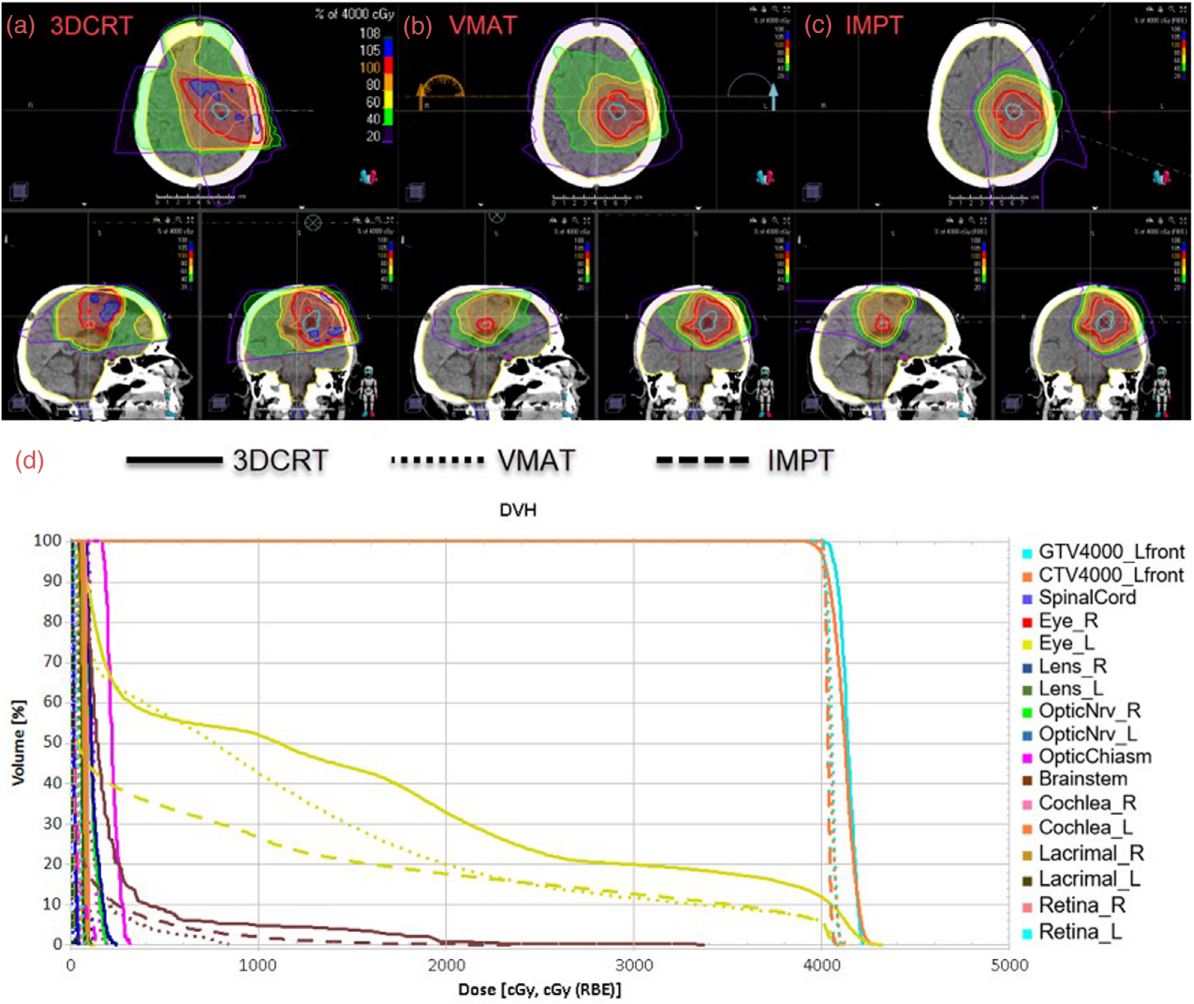
The plan dose distributions for 3DCRT (a), VMAT (b), and IMPT (c). The treatment isocenters are depicted as the cross in a circle in each plan. The dose volume histograms for all three plans are shown in (d).

The plan dose distributions are presented in Figure 1a‐c for 3DCRT, VMAT, and IMPT, with the corresponding dose‐volume histograms shown in Figure 1d.

### 2.2 Out‐of‐field fetal dose measurement for photon plans

**FIGURE 2:**
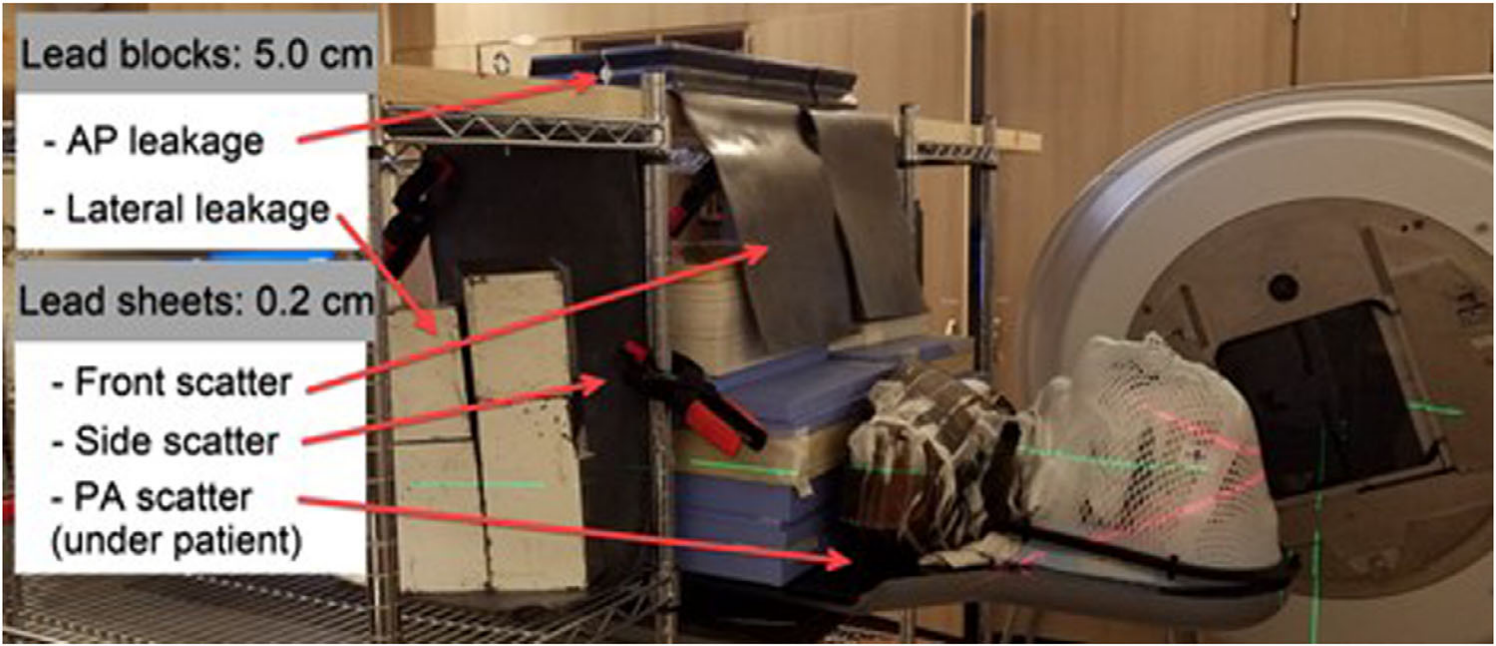
Shielding arrangements for out‐of‐field fetal dose measurement for photon plans.

In pre‐treatment evaluations, OSLDs were positioned on the phantom at various distances from the field edge to measure the out‐of‐field dose for photon plans. Pre‐treatment measurements were conducted both with and without shielding. In the shielding arrangement, as illustrated in Figure 2, 0.2 cm flat lead plates were used to address low‐energy scattered dose, while 5 cm lead blocks were employed to shield against high‐energy gantry head leakage. The pre‐treatment measurements led to the conclusion that the photon plans met the clinical acceptability criteria, particularly when the shielding was applied (Table 1). Observations indicated that the shielding resulted in a reduction in fetal dose by a factor exceeding 2.

**TABLE 1:** Out‐of‐field dose from photon (fetus shielding was utilized) and proton plans, for distances from the field edge.

	Photon (OSLD) mSv/Gy		Proton (Neutrak) mSv/Gy		
Points in phantom, cm from field edge	3DCRT	VMAT	Neutron	Photon	Total
30	0.59	0.51	0.06	0.002	0.06
40	0.30	0.35	0.04	0.001	0.04
50	0.08	0.17	0.01	0.000	0.01

### 2.3 Out‐of‐field fetal dose measurement for proton plans

For pre‐treatment measurements of proton plans, both a WENDI‐II detector and Landauer Neutrak neutron detectors were utilized. The WENDI‐II‐2 detector is particularly advantageous for measuring the out‐of‐field neutron dose in proton therapy due to its broad energy range detection capabilities and high sensitivity. The WENDI‐II measurement is the neutron ambient dose equivalent H^10^, accounting for the physical dose deposited and the quality factor (Q). Measurement uncertainty with WENDI‐II detectors stems from factors like energy response variations, calibration accuracy, but mainly the large size of the detector, which complicates its placement inside anthropomorphic phantoms to estimate fetal doses.

Landauer Neutrak neutron detectors facilitate neutron radiation monitoring through the utilization of CR‐39 and Track Etch technology.^8^ The Neutrak detector is constructed using CR‐39 (allyl diglycol carbonate) and is a solid‐state nuclear track detector that is not responsive to x‐rays, beta particles, or gamma radiation. Neutrak possesses an energy range spanning from 0.25 eV to 40 MeV and offers a dose measurement range from 0.01 to 25 rem (equivalent to 0.1–250 mSv). Measurement uncertainty with Neutrak detectors arises from background noise, calibration discrepancies, detector response variations, and positioning errors. More importantly, careful positioning and adherence to calibration protocols are vital to mitigate uncertainties and ensure accurate neutron dose measurements.^8^ Additionally, its limited sensitivity range can be challenging when using them for plans treating deep sitting tumors.

The highest energy of the proton beam in the IMPT plan for this patient was 120 MeV, the corresponding neutron spectrum from this proton plan thus is outside the Neutrak neutron detector sensitivity range. Figure 2 of TG158^8^ gives the neutron fluence spectrum per unit lethargy per proton Gy to the isocenter for PBS energy of 172 MeV with a measurement location at 1.15 m downstream from the isocenter with the phantom present. In this figure, the amplitude of the dose distribution in the peak area, that is, 172 MeV, represents about a 10‐fold increase, conservatively, compared to the amplitude at the 40 MeV region, which is the Neutrak limited detection. Therefore, in the measurement of the out‐of‐field neutrons from clinical plans, using Neutrak detectors, a coefficient factor of 10 was applied to all readings at about the 40 MeV range to estimate the neutron dose at the 140 MeV range. Readings obtained from Neutrak detectors, and the estimated values were cross‐checked with WENDI‐II detector readings, at places where the bulky WENDI‐II could be placed, with a particular focus on verifying the 10‐fold adjustment factor. Results turned out to be consistent between WENDI‐II and Neutrak. Consequently, Neutrak detectors were utilized at multiple locations around the phantom and in between different phantom layers, leveraging their small size and high sensitivity, and the correction factor was applied to the readings. Neutrak detectors were also employed for in vivo measurements. These detectors were positioned on the patient's body. Consequently, to assess the dose to the fetus within the patient's body, the readings were corrected based on the neutron PDDE, as outlined in AAPM TG158^8^.

### 2.4 On treatment's considerations

Based on the outcomes of the pre‐treatment investigations, proton treatment was chosen for the patient. To mitigate fetal dose from external sources, including in‐room generated secondary particles, the patient was scheduled for the first appointment each morning during treatment. Daily imaging was performed using kV/kV and only minimal necessary cone beam CT (CBCT), with lead aprons placed around the patient's abdomen and pelvis during imaging. Our imaging dose measurements indicated minimal exposure, with KV imaging yielding a fraction of mGy and CBCT approximately 10 mGy. Surface‐guided radiation therapy (SGRT) was employed to ensure precise patient positioning during treatment. Routine weekly quality assurance CT scans (QACTs) were replaced by quality assurance MR imaging (QAMRI) simulations.

During treatment sessions, in vivo measurements were performed on the patient using detectors sensitive to both photon and neutron doses. The calibration point of the detectors was appropriately adjusted based on the neutron percent depth dose equivalent (PDDE), as illustrated in Figure 1 of TG158^8^, to give an estimated fetus dose from the reading at the patient's body. Subsequently, a retrospective analysis of the data was conducted and compared with the data obtained from the pre‐treatment phantom study. A close alignment between the two sets of data, including pre‐treatment data and in vivo results, was observed.

## 3 RESULTS

The results of the pre‐treatment studies demonstrate that proton therapy significantly reduced the out‐of‐field dose to the fetus, as presented in Table 1.

In photon pre‐treatment measurements, the doses measured at 30 cm from the field edge, with shielding and inside the phantom, were 0.59 and 0.51 mSv/Gy for 3DCRT and VMAT, respectively. At distances of 40 cm, doses for 3DCRT and VMAT were measured as 0.30 and 0.35 mSv/Gy, respectively, and at 50 cm, they were measured as 0.08 and 0.17 mSv/Gy. All readings were two‐fold larger without the shielding.

In proton pre‐treatment measurements, the total fetus dose was 0.06, 0.04, and 0.01 mSv/Gy at distances of 30 , 40 , and 50 cm, respectively. Neutron and photon contributions were 0.06 and 0.002 mSv/Gy, 0.04 and 0.001 mSv/Gy, and 0.01 and 0.00 mSv/Gy, respectively. All detected neutron doses were fast neutrons, with no thermal neutrons detected. All readings were done at a depth representing the fetus in the patient; therefore, PDDE was not applied.

## 4 DISCUSSION

The use of radiation therapy during pregnancy for cancer treatment presents a complex challenge. The guiding principle for minimizing fetal out‐of‐field radiation exposure is the ALARA (As Low As Reasonably Achievable) principle, while ensuring effective radiation treatment for the mother. One of the practical challenges in the treatment of pregnant patients with proton therapy is the wide energy range of the secondary neutrons. The biological effect of neutrons is energy dependent. Therefore, the current work and similar dosimetric studies of out‐of‐field radiation exposure to the fetus should be considered with its involved uncertainties.^31^, ^32^ Our study demonstrates that IMPT with the PBS system was the superior choice over 3DCRT and VMAT for the patient's treatment, ensuring both effective treatment and the safety of the baby.

Plans for pregnant patients receiving proton therapy can be optimized such that, while holding a clinically acceptable plan for the mother, the out‐of‐field dose exposure to the fetus is minimized. Specific considerations include avoiding high‐energy beams aligned with the fetus and minimizing neutron scatter by limiting the beamline components. For disease sites such as the brain and head and neck, the fetal doses can be below acceptable threshold levels; however, when treating more challenging cases, such as craniospinal irradiation, it is essential to consider dosimetric uncertainties to ensure that fetal doses remain within acceptable limits.^22^, ^33^ Further research is needed to quantify the impact of different planning parameters on fetal radiation exposure (work in progress).

While abdominal shielding presents challenges in design and setup for every photon treatment fraction, its usage has been observed due to its perceived benefits. In proton PBS, patient‐specific shielding, which protects fetus from the predominant out‐of‐field neutron dose, should be evaluated case‐by‐case, though generally speaking, evidence suggests it has no to very limited benefit to the patient. An exception to the limited benefit of patient‐specific shielding in proton PBS would be during the imaging of the proton patient.

The findings of our study align with previous research efforts investigating fetal radiation dose exposure during radiotherapy. Hopfensperger et al.^34^ utilized the WENDI‐II detector to measure fetal doses, indicating significantly lower exposure levels ranging from 22.5 to 33.2 µSv/Gy compared to photon‐RT. Monte Carlo simulations have further contributed to our understanding, with studies by Geng et al.^14^ and De Saint Hubert et al.^35^ highlighting the importance of computational modeling in estimating fetal doses, emphasizing the variability based on gestational age and anatomical positioning. Moreover, clinical cases described by Yeom et al.,^36^ Wang et al.,^23^ and Kalbasi et al.^22^ underscore the critical need for precise treatment planning to minimize fetal exposure, showcasing successful strategies to mitigate risks while ensuring favorable outcomes for both mother and child. Collectively, these investigations reinforce the significance of tailored approaches in radiotherapy to safeguard fetal well‐being, emphasizing the consistency of our findings with prior research efforts aimed at optimizing therapeutic efficacy while prioritizing maternal and fetal safety.

## 5 CONCLUSION

The findings of this study are consistent with previous studies that have shown the superiority of proton therapy in reducing the dose to normal tissues.^20^, ^21^, ^22^, ^23^, ^24^, ^25^ Our study demonstrates that intensity‐modulated proton therapy (IMPT) has the potential to significantly minimize fetal dose exposure during radiation therapy for pregnant patients with brain tumors, even when accounting for neutron dose generated from protons. These results underscore the potential benefits of proton therapy in the management of brain tumors in pregnant patients and emphasize the importance of individualized treatment planning to minimize the risk of harm to the developing fetus while achieving optimal treatment outcomes for the mother. Implementation of a clinically advanced beam delivery system, combined with individualized neutron‐reduction treatment plans, further mitigates potential risks associated with radiation therapy during pregnancy, ensuring an optimal outcome for both the mother and the developing fetus.

## AUTHOR CONTRIBUTIONS

Proton plan was done by Grayden MacLennan. Photon plans were done by Ashkan Parniani and Erin Murdoch. Daniel Kim was the MD for the case study. Ashish Chawla was consulting MD for the clinical case. Michael Taylor and Lienard Chang were Health Physics personnels and prepared the shielding. Peng Wang and Jiajin Fan initiated the work and were providing consultants throughout the work. Xing Li did the formatting of the work. Xing Li, Kuan Ling Chen, and Peng Wang did the proofreading and provided comments for completeness of the work. Robabeh Rahimi did the study and designed the photon shielding and wrote the manuscript.

## CONFLICT OF INTEREST STATEMENT

The authors declare no conflicts of interest.
